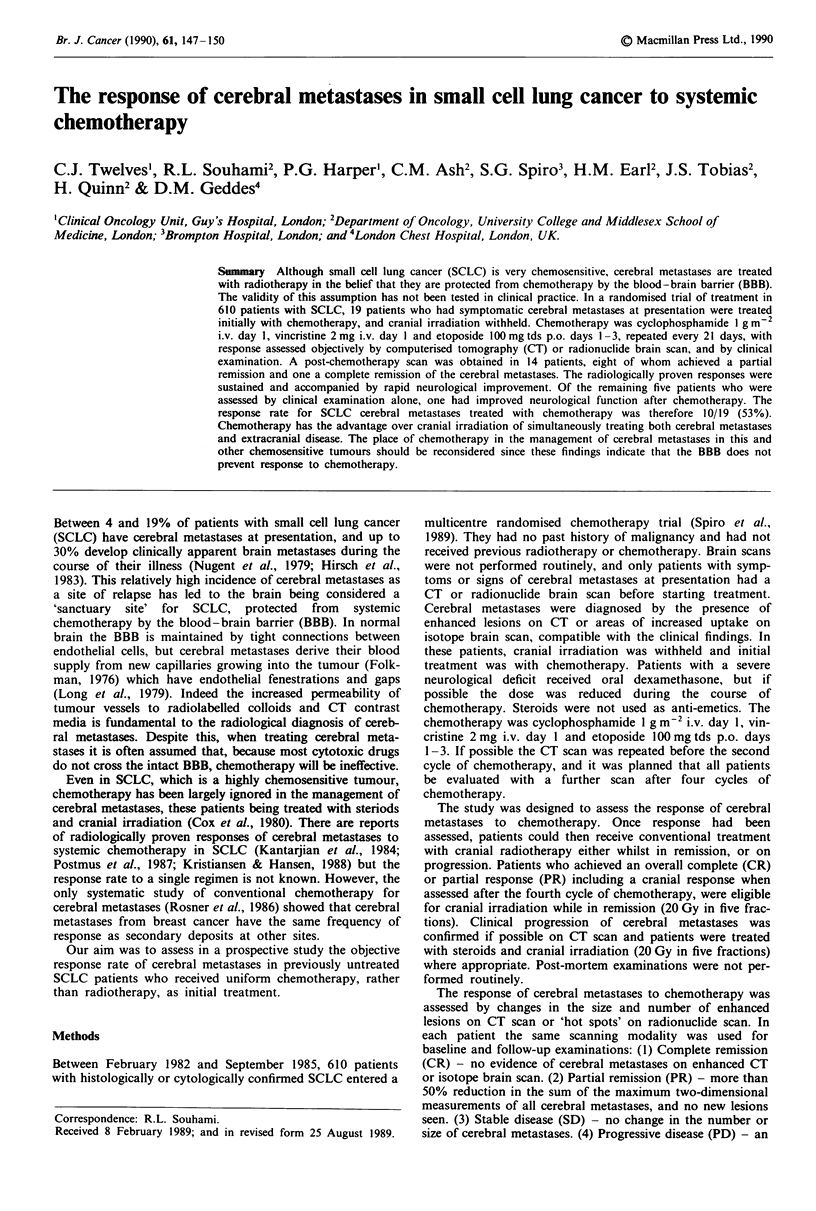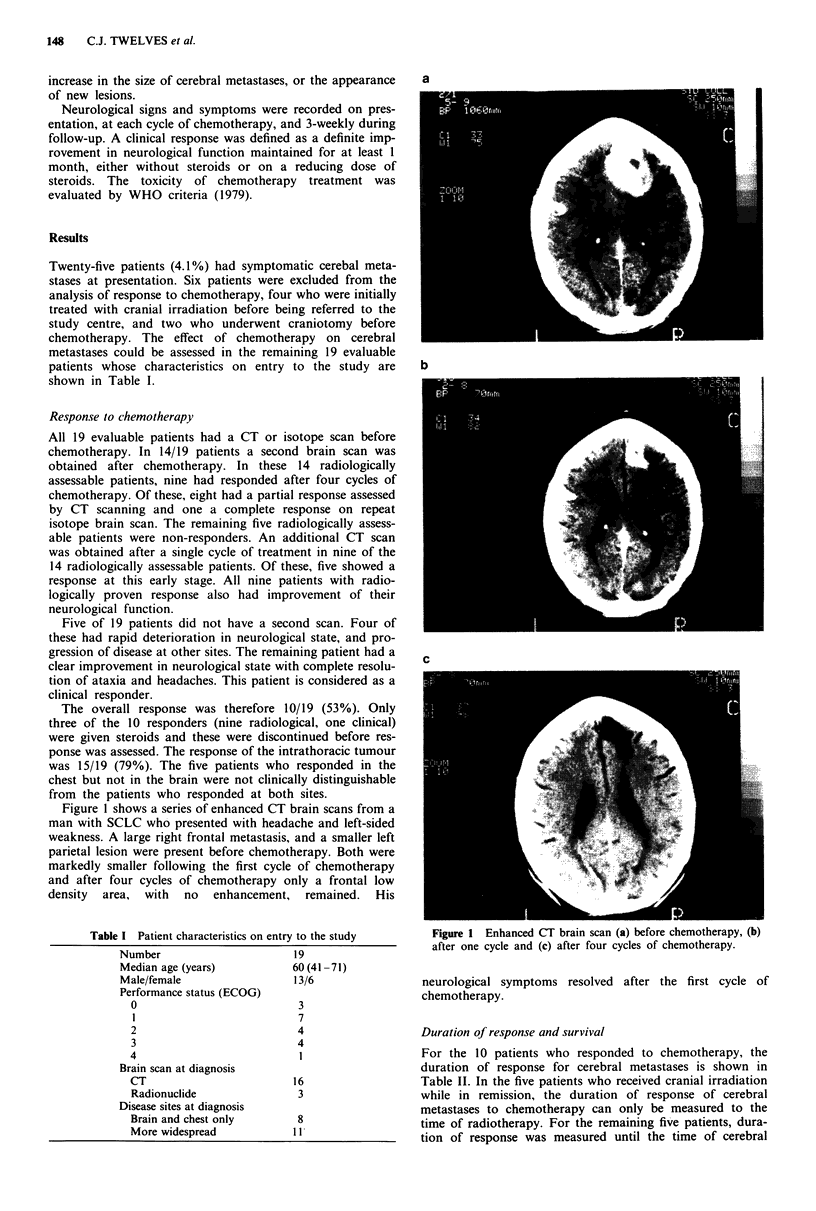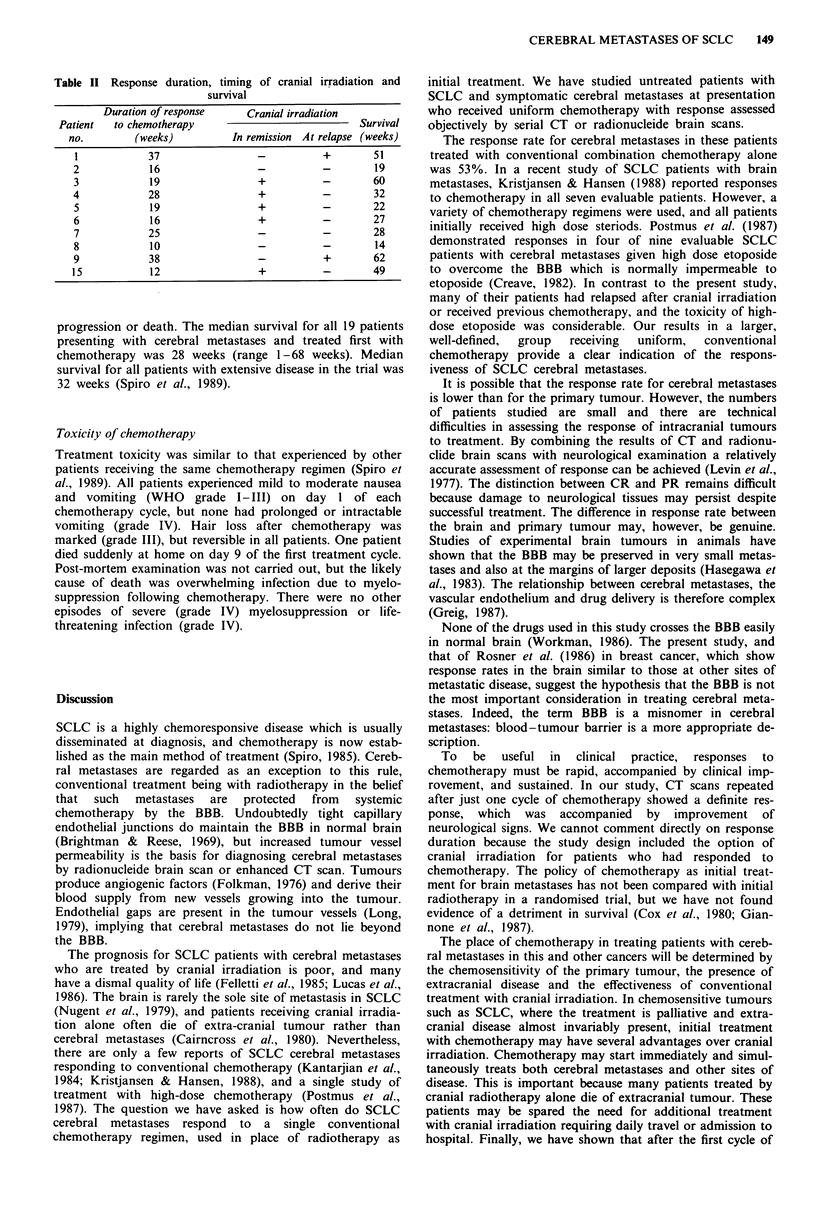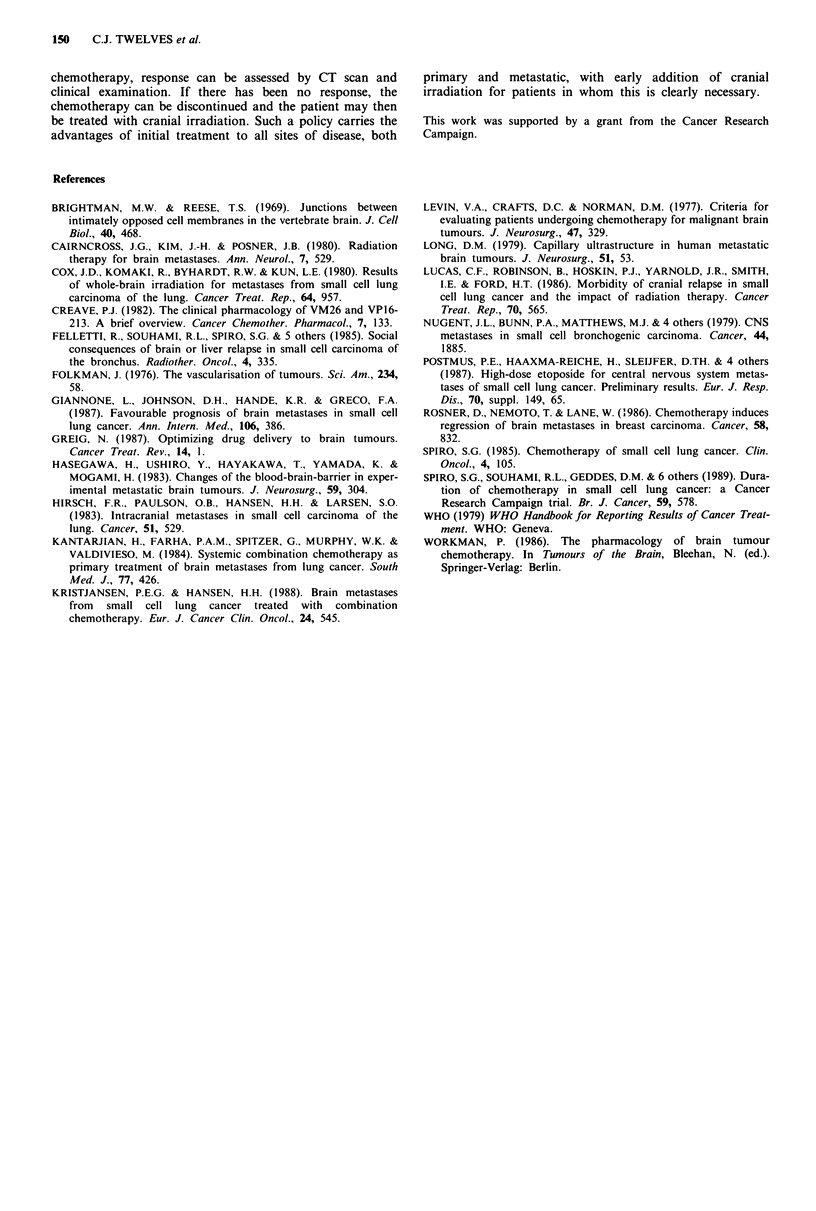# The response of cerebral metastases in small cell lung cancer to systemic chemotherapy.

**DOI:** 10.1038/bjc.1990.30

**Published:** 1990-01

**Authors:** C. J. Twelves, R. L. Souhami, P. G. Harper, C. M. Ash, S. G. Spiro, H. M. Earl, J. S. Tobias, H. Quinn, D. M. Geddes

**Affiliations:** Clinical Oncology Unit, Guy's Hospital, London, UK.

## Abstract

**Images:**


Br. J. Cancer (1990), 61, 147-150 © Macmillan Press Ltd., 1990

### The response of cerebral metastases in small cell lung cancer to systemic chemotherapy

C.J. Twelves[1], R.L. Souhami[2], P.G. Harper[1], C.M. Ash[2], S.G. Spiro[3], H.M. Earl[2], J.S. Tobias[2], H. Quinn[2] & D.M. Geddes[4]

[1]*Clinical Oncology Unit, Guy's Hospital, London;* [2]*Department of Oncology, University College and Middlesex School of Medicine, London;* [3]*Brompton Hospital, London; and* [4]*London Chest Hospital, London, UK.*

**Summary** Although small cell lung cancer (SCLC) is very chemosensitive, cerebral metastases are treated with radiotherapy in the belief that they are protected from chemotherapy by the blood-brain barrier (BBB). The validity of this assumption has not been tested in clinical practice. In a randomised trial of treatment in 610 patients with SCLC, 19 patients who had symptomatic cerebral metastases at presentation were treated initially with chemotherapy, and cranial irradiation withheld. Chemotherapy was cyclophosphamide 1 g m$^{-2}$ i.v. day 1, vincristine 2 mg i.v. day 1 and etoposide 100 mg tds p.o. days 1-3, repeated every 21 days, with response assessed objectively by computerised tomography (CT) or radionuclide brain scan, and by clinical examination. A post-chemotherapy scan was obtained in 14 patients, eight of whom achieved a partial remission and one a complete remission of the cerebral metastases. The radiologically proven responses were sustained and accompanied by rapid neurological improvement. Of the remaining five patients who were assessed by clinical examination alone, one had improved neurological function after chemotherapy. The response rate for SCLC cerebral metastases treated with chemotherapy was therefore 10/19 (53%). Chemotherapy has the advantage over cranial irradiation of simultaneously treating both cerebral metastases and extracranial disease. The place of chemotherapy in the management of cerebral metastases in this and other chemosensitive tumours should be reconsidered since these findings indicate that the BBB does not prevent response to chemotherapy.

Between 4 and 19% of patients with small cell lung cancer (SCLC) have cerebral metastases at presentation, and up to 30% develop clinically apparent brain metastases during the course of their illness (Nugent *et al.*, 1979; Hirsch *et al.*, 1983). This relatively high incidence of cerebral metastases as a site of relapse has led to the brain being considered a 'sanctuary site' for SCLC, protected from systemic chemotherapy by the blood-brain barrier (BBB). In normal brain the BBB is maintained by tight connections between endothelial cells, but cerebral metastases derive their blood supply from new capillaries growing into the tumour (Folkman, 1976) which have endothelial fenestrations and gaps (Long *et al.*, 1979). Indeed the increased permeability of tumour vessels to radiolabelled colloids and CT contrast media is fundamental to the radiological diagnosis of cerebral metastases. Despite this, when treating cerebral metastases it is often assumed that, because most cytotoxic drugs do not cross the intact BBB, chemotherapy will be ineffective.

Even in SCLC, which is a highly chemosensitive tumour, chemotherapy has been largely ignored in the management of cerebral metastases, these patients being treated with steriods and cranial irradiation (Cox *et al.*, 1980). There are reports of radiologically proven responses of cerebral metastases to systemic chemotherapy in SCLC (Kantarjian *et al.*, 1984; Postmus *et al.*, 1987; Kristiansen & Hansen, 1988) but the response rate to a single regimen is not known. However, the only systematic study of conventional chemotherapy for cerebral metastases (Rosner *et al.*, 1986) showed that cerebral metastases from breast cancer have the same frequency of response as secondary deposits at other sites.

Our aim was to assess in a prospective study the objective response rate of cerebral metastases in previously untreated SCLC patients who received uniform chemotherapy, rather than radiotherapy, as initial treatment.

### Methods

Between February 1982 and September 1985, 610 patients with histologically or cytologically confirmed SCLC entered a multicentre randomised chemotherapy trial (Spiro *et al.*, 1989). They had no past history of malignancy and had not received previous radiotherapy or chemotherapy. Brain scans were not performed routinely, and only patients with symptoms or signs of cerebral metastases at presentation had a CT or radionuclide brain scan before starting treatment. Cerebral metastases were diagnosed by the presence of enhanced lesions on CT or areas of increased uptake on isotope brain scan, compatible with the clinical findings. In these patients, cranial irradiation was withheld and initial treatment was with chemotherapy. Patients with a severe neurological deficit received oral dexamethasone, but if possible the dose was reduced during the course of chemotherapy. Steroids were not used as anti-emetics. The chemotherapy was cyclophosphamide 1 g m$^{-2}$ i.v. day 1, vincristine 2 mg i.v. day 1 and etoposide 100 mg tds p.o. days 1-3. If possible the CT scan was repeated before the second cycle of chemotherapy, and it was planned that all patients be evaluated with a further scan after four cycles of chemotherapy.

The study was designed to assess the response of cerebral metastases to chemotherapy. Once response had been assessed, patients could then receive conventional treatment with cranial radiotherapy either whilst in remission, or on progression. Patients who achieved an overall complete (CR) or partial response (PR) including a cranial response when assessed after the fourth cycle of chemotherapy, were eligible for cranial irradiation while in remission (20 Gy in five fractions). Clinical progression of cerebral metastases was confirmed if possible on CT scan and patients were treated with steroids and cranial irradiation (20 Gy in five fractions) where appropriate. Post-mortem examinations were not performed routinely.

The response of cerebral metastases to chemotherapy was assessed by changes in the size and number of enhanced lesions on CT scan or 'hot spots' on radionuclide scan. In each patient the same scanning modality was used for baseline and follow-up examinations: (1) Complete remission (CR) - no evidence of cerebral metastases on enhanced CT or isotope brain scan. (2) Partial remission (PR) - more than 50% reduction in the sum of the maximum two-dimensional measurements of all cerebral metastases, and no new lesions seen. (3) Stable disease (SD) - no change in the number or size of cerebral metastases. (4) Progressive disease (PD) - an

Correspondence: R.L. Souhami.
Received 8 February 1989; and in revised form 25 August 1989.

increase in the size of cerebral metastases, or the appearance of new lesions.

Neurological signs and symptoms were recorded on presentation, at each cycle of chemotherapy, and 3-weekly during follow-up. A clinical response was defined as a definite improvement in neurological function maintained for at least 1 month, either without steroids or on a reducing dose of steroids. The toxicity of chemotherapy treatment was evaluated by WHO criteria (1979).

### Results

Twenty-five patients (4.1%) had symptomatic cerebal metastases at presentation. Six patients were excluded from the analysis of response to chemotherapy, four who were initially treated with cranial irradiation before being referred to the study centre, and two who underwent craniotomy before chemotherapy. The effect of chemotherapy on cerebral metastases could be assessed in the remaining 19 evaluable patients whose characteristics on entry to the study are shown in Table I.

### *Response to chemotherapy*

All 19 evaluable patients had a CT or isotope scan before chemotherapy. In 14/19 patients a second brain scan was obtained after chemotherapy. In these 14 radiologically assessable patients, nine had responded after four cycles of chemotherapy. Of these, eight had a partial response assessed by CT scanning and one a complete response on repeat isotope brain scan. The remaining five radiologically assessable patients were non-responders. An additional CT scan was obtained after a single cycle of treatment in nine of the 14 radiologically assessable patients. Of these, five showed a response at this early stage. All nine patients with radiologically proven response also had improvement of their neurological function.

Five of 19 patients did not have a second scan. Four of these had rapid deterioration in neurological state, and progression of disease at other sites. The remaining patient had a clear improvement in neurological state with complete resolution of ataxia and headaches. This patient is considered as a clinical responder.

The overall response was therefore 10/19 (53%). Only three of the 10 responders (nine radiological, one clinical) were given steroids and these were discontinued before response was assessed. The response of the intrathoracic tumour was 15/19 (79%). The five patients who responded in the chest but not in the brain were not clinically distinguishable from the patients who responded at both sites.

Figure 1 shows a series of enhanced CT brain scans from a man with SCLC who presented with headache and left-sided weakness. A large right frontal metastasis, and a smaller left parietal lesion were present before chemotherapy. Both were markedly smaller following the first cycle of chemotherapy and after four cycles of chemotherapy only a frontal low density area, with no enhancement, remained. His

**Table I** Patient characteristics on entry to the study

| | |
|---|---|
| Number | 19 |
| Median age (years) | 60 (41–71) |
| Male/female | 13/6 |
| Performance status (ECOG) | |
| 0 | 3 |
| 1 | 7 |
| 2 | 4 |
| 3 | 4 |
| 4 | 1 |
| Brain scan at diagnosis | |
| CT | 16 |
| Radionuclide | 3 |
| Disease sites at diagnosis | |
| Brain and chest only | 8 |
| More widespread | 11 |

a b c

**Figure 1** Enhanced CT brain scan (**a**) before chemotherapy, (**b**) after one cycle and (**c**) after four cycles of chemotherapy.

neurological symptoms resolved after the first cycle of chemotherapy.

### *Duration of response and survival*

For the 10 patients who responded to chemotherapy, the duration of response for cerebral metastases is shown in Table II. In the five patients who received cranial irradiation while in remission, the duration of response of cerebral metastases to chemotherapy can only be measured to the time of radiotherapy. For the remaining five patients, duration of response was measured until the time of cerebral

**Table II** Response duration, timing of cranial irradiation and survival

| Patient no. | Duration of response to chemotherapy (weeks) | Cranial irradiation In remission | Cranial irradiation At relapse | Survival (weeks) |
|---|---|---|---|---|
| 1 | 37 | – | + | 51 |
| 2 | 16 | – | – | 19 |
| 3 | 19 | + | – | 60 |
| 4 | 28 | + | – | 32 |
| 5 | 19 | + | – | 22 |
| 6 | 16 | + | – | 27 |
| 7 | 25 | – | – | 28 |
| 8 | 10 | – | – | 14 |
| 9 | 38 | – | + | 62 |
| 15 | 12 | + | – | 49 |

progression or death. The median survival for all 19 patients presenting with cerebral metastases and treated first with chemotherapy was 28 weeks (range 1–68 weeks). Median survival for all patients with extensive disease in the trial was 32 weeks (Spiro *et al.*, 1989).

*Toxicity of chemotherapy*

Treatment toxicity was similar to that experienced by other patients receiving the same chemotherapy regimen (Spiro *et al.*, 1989). All patients experienced mild to moderate nausea and vomiting (WHO grade I–III) on day 1 of each chemotherapy cycle, but none had prolonged or intractable vomiting (grade IV). Hair loss after chemotherapy was marked (grade III), but reversible in all patients. One patient died suddenly at home on day 9 of the first treatment cycle. Post-mortem examination was not carried out, but the likely cause of death was overwhelming infection due to myelosuppression following chemotherapy. There were no other episodes of severe (grade IV) myelosuppression or life-threatening infection (grade IV).

### Discussion

SCLC is a highly chemoresponsive disease which is usually disseminated at diagnosis, and chemotherapy is now established as the main method of treatment (Spiro, 1985). Cerebral metastases are regarded as an exception to this rule, conventional treatment being with radiotherapy in the belief that such metastases are protected from systemic chemotherapy by the BBB. Undoubtedly tight capillary endothelial junctions do maintain the BBB in normal brain (Brightman & Reese, 1969), but increased tumour vessel permeability is the basis for diagnosing cerebral metastases by radionucleide brain scan or enhanced CT scan. Tumours produce angiogenic factors (Folkman, 1976) and derive their blood supply from new vessels growing into the tumour. Endothelial gaps are present in the tumour vessels (Long, 1979), implying that cerebral metastases do not lie beyond the BBB.

The prognosis for SCLC patients with cerebral metastases who are treated by cranial irradiation is poor, and many have a dismal quality of life (Felletti *et al.*, 1985; Lucas *et al.*, 1986). The brain is rarely the sole site of metastasis in SCLC (Nugent *et al.*, 1979), and patients receiving cranial irradiation alone often die of extra-cranial tumour rather than cerebral metastases (Cairncross *et al.*, 1980). Nevertheless, there are only a few reports of SCLC cerebral metastases responding to conventional chemotherapy (Kantarjian *et al.*, 1984; Kristjansen & Hansen, 1988), and a single study of treatment with high-dose chemotherapy (Postmus *et al.*, 1987). The question we have asked is how often do SCLC cerebral metastases respond to a single conventional chemotherapy regimen, used in place of radiotherapy as initial treatment. We have studied untreated patients with SCLC and symptomatic cerebral metastases at presentation who received uniform chemotherapy with response assessed objectively by serial CT or radionucleide brain scans.

The response rate for cerebral metastases in these patients treated with conventional combination chemotherapy alone was 53%. In a recent study of SCLC patients with brain metastases, Kristjansen & Hansen (1988) reported responses to chemotherapy in all seven evaluable patients. However, a variety of chemotherapy regimens were used, and all patients initially received high dose steriods. Postmus *et al.* (1987) demonstrated responses in four of nine evaluable SCLC patients with cerebral metastases given high dose etoposide to overcome the BBB which is normally impermeable to etoposide (Creave, 1982). In contrast to the present study, many of their patients had relapsed after cranial irradiation or received previous chemotherapy, and the toxicity of high-dose etoposide was considerable. Our results in a larger, well-defined, group receiving uniform, conventional chemotherapy provide a clear indication of the responsiveness of SCLC cerebral metastases.

It is possible that the response rate for cerebral metastases is lower than for the primary tumour. However, the numbers of patients studied are small and there are technical difficulties in assessing the response of intracranial tumours to treatment. By combining the results of CT and radionuclide brain scans with neurological examination a relatively accurate assessment of response can be achieved (Levin *et al.*, 1977). The distinction between CR and PR remains difficult because damage to neurological tissues may persist despite successful treatment. The difference in response rate between the brain and primary tumour may, however, be genuine. Studies of experimental brain tumours in animals have shown that the BBB may be preserved in very small metastases and also at the margins of larger deposits (Hasegawa *et al.*, 1983). The relationship between cerebral metastases, the vascular endothelium and drug delivery is therefore complex (Greig, 1987).

None of the drugs used in this study crosses the BBB easily in normal brain (Workman, 1986). The present study, and that of Rosner *et al.* (1986) in breast cancer, which show response rates in the brain similar to those at other sites of metastatic disease, suggest the hypothesis that the BBB is not the most important consideration in treating cerebral metastases. Indeed, the term BBB is a misnomer in cerebral metastases: blood–tumour barrier is a more appropriate description.

To be useful in clinical practice, responses to chemotherapy must be rapid, accompanied by clinical improvement, and sustained. In our study, CT scans repeated after just one cycle of chemotherapy showed a definite response, which was accompanied by improvement of neurological signs. We cannot comment directly on response duration because the study design included the option of cranial irradiation for patients who had responded to chemotherapy. The policy of chemotherapy as initial treatment for brain metastases has not been compared with initial radiotherapy in a randomised trial, but we have not found evidence of a detriment in survival (Cox *et al.*, 1980; Giannone *et al.*, 1987).

The place of chemotherapy in treating patients with cerebral metastases in this and other cancers will be determined by the chemosensitivity of the primary tumour, the presence of extracranial disease and the effectiveness of conventional treatment with cranial irradiation. In chemosensitive tumours such as SCLC, where the treatment is palliative and extracranial disease almost invariably present, initial treatment with chemotherapy may have several advantages over cranial irradiation. Chemotherapy may start immediately and simultaneously treats both cerebral metastases and other sites of disease. This is important because many patients treated by cranial radiotherapy alone die of extracranial tumour. These patients may be spared the need for additional treatment with cranial irradiation requiring daily travel or admission to hospital. Finally, we have shown that after the first cycle of chemotherapy, response can be assessed by CT scan and clinical examination. If there has been no response, the chemotherapy can be discontinued and the patient may then be treated with cranial irradiation. Such a policy carries the advantages of initial treatment to all sites of disease, both primary and metastatic, with early addition of cranial irradiation for patients in whom this is clearly necessary.

This work was supported by a grant from the Cancer Research Campaign.

### References

BRIGHTMAN, M.W. & REESE, T.S. (1969). Junctions between intimately opposed cell membranes in the vertebrate brain. *J. Cell Biol.*, **40**, 468.

CAIRNCROSS, J.G., KIM, J.-H. & POSNER, J.B. (1980). Radiation therapy for brain metastases. *Ann. Neurol.*, **7**, 529.

COX, J.D., KOMAKI, R., BYHARDT, R.W. & KUN, L.E. (1980). Results of whole-brain irradiation for metastases from small cell lung carcinoma of the lung. *Cancer Treat. Rep.*, **64**, 957.

CREAVE, P.J. (1982). The clinical pharmacology of VM26 and VP16-213. A brief overview. *Cancer Chemother. Pharmacol.*, **7**, 133.

FELLETTI, R., SOUHAMI, R.L., SPIRO, S.G. & 5 others (1985). Social consequences of brain or liver relapse in small cell carcinoma of the bronchus. *Radiother. Oncol.*, **4**, 335.

FOLKMAN, J. (1976). The vascularisation of tumours. *Sci. Am.*, **234**, 58.

GIANNONE, L., JOHNSON, D.H., HANDE, K.R. & GRECO, F.A. (1987). Favourable prognosis of brain metastases in small cell lung cancer. *Ann. Intern. Med.*, **106**, 386.

GREIG, N. (1987). Optimizing drug delivery to brain tumours. *Cancer Treat. Rev.*, **14**, 1.

HASEGAWA, H., USHIRO, Y., HAYAKAWA, T., YAMADA, K. & MOGAMI, H. (1983). Changes of the blood-brain-barrier in experimental metastatic brain tumours. *J. Neurosurg.*, **59**, 304.

HIRSCH, F.R., PAULSON, O.B., HANSEN, H.H. & LARSEN, S.O. (1983). Intracranial metastases in small cell carcinoma of the lung. *Cancer*, **51**, 529.

KANTARJIAN, H., FARHA, P.A.M., SPITZER, G., MURPHY, W.K. & VALDIVIESO, M. (1984). Systemic combination chemotherapy as primary treatment of brain metastases from lung cancer. *South Med. J.*, **77**, 426.

KRISTJANSEN, P.E.G. & HANSEN, H.H. (1988). Brain metastases from small cell lung cancer treated with combination chemotherapy. *Eur. J. Cancer Clin. Oncol.*, **24**, 545.

LEVIN, V.A., CRAFTS, D.C. & NORMAN, D.M. (1977). Criteria for evaluating patients undergoing chemotherapy for malignant brain tumours. *J. Neurosurg.*, **47**, 329.

LONG, D.M. (1979). Capillary ultrastructure in human metastatic brain tumours. *J. Neurosurg.*, **51**, 53.

LUCAS, C.F., ROBINSON, B., HOSKIN, P.J., YARNOLD, J.R., SMITH, I.E. & FORD, H.T. (1986). Morbidity of cranial relapse in small cell lung cancer and the impact of radiation therapy. *Cancer Treat. Rep.*, **70**, 565.

NUGENT, J.L., BUNN, P.A., MATTHEWS, M.J. & 4 others (1979). CNS metastases in small cell bronchogenic carcinoma. *Cancer*, **44**, 1885.

POSTMUS, P.E., HAAXMA-REICHE, H., SLEIJFER, D.TH. & 4 others (1987). High-dose etoposide for central nervous system metastases of small cell lung cancer. Preliminary results. *Eur. J. Resp. Dis.*, **70**, suppl. 149, 65.

ROSNER, D., NEMOTO, T. & LANE, W. (1986). Chemotherapy induces regression of brain metastases in breast carcinoma. *Cancer*, **58**, 832.

SPIRO, S.G. (1985). Chemotherapy of small cell lung cancer. *Clin. Oncol.*, **4**, 105.

SPIRO, S.G., SOUHAMI, R.L., GEDDES, D.M. & 6 others (1989). Duration of chemotherapy in small cell lung cancer: a Cancer Research Campaign trial. *Br. J. Cancer*, **59**, 578.

WHO (1979) *WHO Handbook for Reporting Results of Cancer Treatment*. WHO: Geneva.

WORKMAN, P. (1986). The pharmacology of brain tumour chemotherapy. In *Tumours of the Brain*, Bleehan, N. (ed.). Springer-Verlag: Berlin.